# A case of permanent vision loss due to a carotidcavernous fistula: importance of early diagnosis

**DOI:** 10.5935/0004-2749.20220054

**Published:** 2025-08-21

**Authors:** Ítalo Pena de Oliveira, Francyne Veiga Reis Cyrino, Sheila Andrade de Paula Cecchetti, Carlos Yuji Nunomura, Patricia Mitiko Santello Akaishi, Rodrigo Jorge

**Affiliations:** 1 Setor de Oftalmologia, Hospital das Clínicas, Faculdade de Medicina de Ribeirão Preto, Universidade de São Paulo, Ribeirão Preto, SP, Brazil; 2 Faculdade de Medicina de Ribeirão Preto, Universidade de São Paulo, Ribeirão Preto, SP, Brazil

**Keywords:** Carotid-cavernous fistula, Orbital cellulitis, Orbital disease, Visual loss, Endovascular Procedures, Humans, Case reports, Fístula carótidocavernosa, Celulite orbitária, Doença orbitária, Perda visual, Procedimentos endovasculares, Humanos, Relatos de casos

## Abstract

A 97-year-old female presented with spontaneous acute-onset palpebral hyperemia and edema of the right eye that had progressively worsen over the previous three days. These signs did not suggest possible carotid-cavernous fistula until a second examination 72 h later, during which the patient exhibited significant progression. Despite embolization, the patient exhibited sustained corneal edema, clots, and turbidity in the aqueous humor, which resulted in permanent visual loss. A greater level of clinical suspicion for possible carotid-cavernous fistula is warranted on initial presentation of palpebral hyperemia and edema to prevent possible irreversible vision loss.

## Abstract (pt)

O objetivo deste relato é apresentar o caso de uma paciente de 97 anos com início agudo e espontâneo de hiperemia e edema palpebral. Estes sinais não levaram a uma suspeita diagnóstica de fístula carótido-cavernosa até um segundo momento, quando a paciente apresentou progressão importante do quadro clínico. Apesar da realização de tratamento efetivo com embolização da fístula, a paciente manteve alterações oculares como edema de córnea, coágulos e turvação no humor aquoso, e manteve perda visual definitiva.

## INTRODUCTION

Carotid-cavernous fistula (CCF) is an infrequent condition with variable severity arising from aberrant vascular communication between the internal carotid artery (ICA or branches) and/or branches of the external carotid artery with the cavernous sinus (CS). This aberrant communication (shunt) promotes an abrupt change in the direction and distribution of cerebral-orbital blood flow^(1-3)^.

Clinical signs range from mild paresis and dysfunction of cranial nerves III (oculomotor nerve), IV (trochlear nerve), V1 (ophthalmic nerve), V2 (maxillary nerve), and VI (abducens nerve: 85%), to orbital congestion (chemosis and venular dilatation), ocular bruit, and pulsatile proptosis (the classical triad)^(2)^. Other signs and symptoms include diplopia (observed in 50%-68% of cases), ophthalmoplegias, episcleral venular dilatation with arterialization, unilateral glaucoma (in 50%-83% of cases) with asymmetric pulse amplitude during tonometry, papilledema, optic neuritis, intracranial hemorrhage, and epistaxis, among other symptoms^(4)^. CCF can be classified based on shunt anatomy, hemodynamic properties, or etiology, specifically whether onset is spontaneous (30% of cases) or results from trauma (70% of cases)^(2)^.

The aim of the present report is to present a case of CCF in an older female resulting in permanent vision loss despite treatment and to discuss the importance of timely recognition, diagnosis, and treatment of this condition.

## CASE REPORT

**Figure 1:**
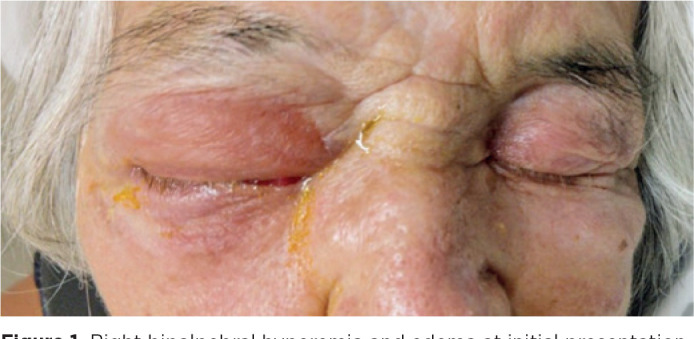
Right bipalpebral hyperemia and edema at initial presentation.

A 97-year-old female was admitted to the emergency department of Hospital das Clínicas da Universidade de São Paulo/USP Ribeirão Preto, with acute-onset spontaneous palpebral hyperemia and edema of the right eye (OD) that had worsened progressively over the past 3 days (Figure 1). She reported well-controlled systemic arterial hypertension and previous cataract surgery on the same eye three months before symptom onset but no other ophthalmological disease history. Ophthalmological evaluation of the OD revealed uncorrected visual acuity (UCVA) for hand movements, reduced direct light reflex, relative afferent pupillary defect, palpebral hyperemia, edema, and chemosis. In the left eye (OE), which was also pseudophacic, UCVA was 20/30 and pupillary reflex was normal. Intra-ocular pressure (IOP) was also slightly higher in the OD (16 mmHg) than the OE (12 mmHg). No proptosis or change in motricity was observed at initial presentation and fundoscopy was not performed. Further, tomography did not detect any orbital abnormalities.

**Figure 2:**
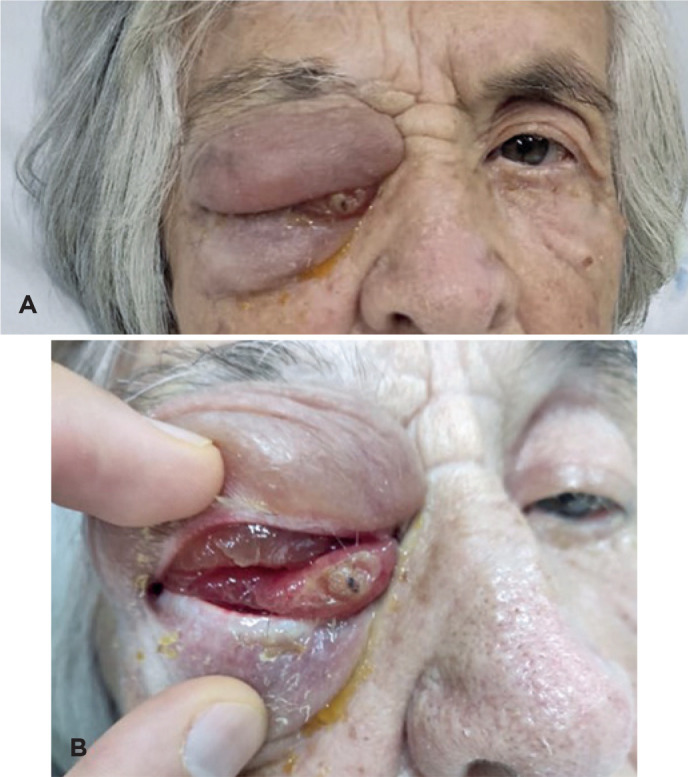
Worsening of bipalpebral edema (A) and chemosis (B) hampering appropriate ocular evaluation.

As no orbital abnormalities were observed by tomography, the tentative diagnosis was pre-septal cellulitis, for which oral and topical antibiotics and warm compress were prescribed. Seventy-two hours later, however, the patient again sought medical care due to worsening ocular symptoms (Figure 2) as well as an episode of spontaneous convulsive tonic-clonic seizures. There were no other signs of infection or systemic involvement, but ophthalmological re-evaluation showed worsening visual acuity (from hands movements to light perception), chemosis, and eyelid edema, which prevented opening of the eyelids and visualization of the bulbus oculi.

**Figure 3:**
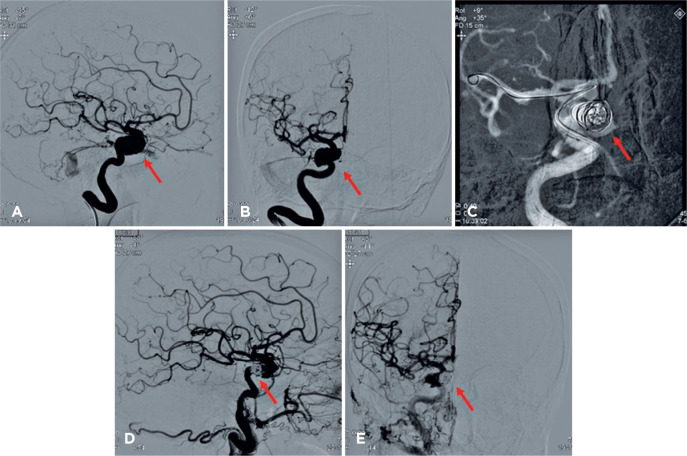
(A) and (B) Cerebral angiography showing a ruptured saccular aneurysm in the internal carotid artery (arrow). (C) ICA catheterization and microcoil introduction for fistula embolization (arrow). (D) and (E) Angiography after embolization showing elimination of the fistula.

**Figure 4:**
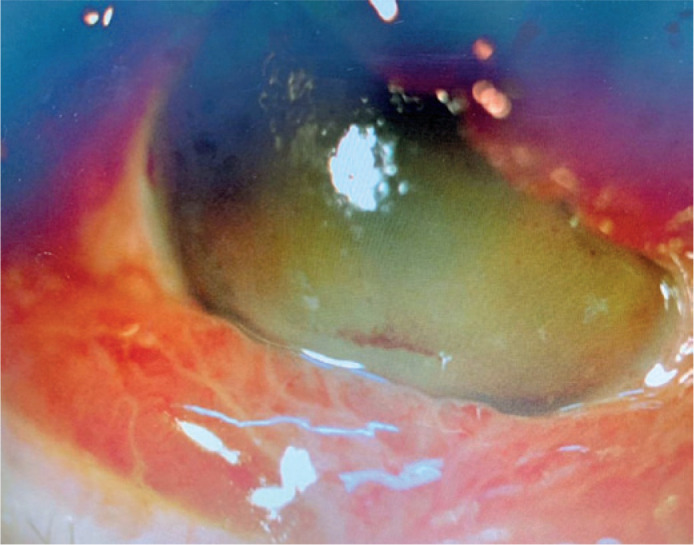
Ocular region after endovascular treatment.

The patient received contrast tomography of the orbit, which revealed dilatation of the superior ophthalmic vein, while arteriography revealing a ruptured saccular aneurysm in the intracavernous portion of the right internal carotid that induced an arteriovenous fistula of the CS (Figure 3A-B). Together with the Neurology and Interventionist Radiology team, it was decided to perform endovascular closure of the fistula (Figure 3C). Anomalous communication was successfully treated (Figure 3D-E), which reduced the eyelid edema and chemosis (Figure 4). Even with treatment, however, corneal edema, hyphema, and vitreous hemorrhage were (4+/4+) on ocular ultrasound, and RE UCVA decreased to near complete absence of light perception. At a 3-month follow-up examination, there was partial improvement of corneal edema and hyphema but no improvement in VA.

## DISCUSSION

Spontaneous direct CCF accounts for about 30% of all fistula cases and is more frequent in middle-aged or older females with no history of eye trauma^(1,2)^. The pathogenesis of spontaneous fistula is still unclear. It is believed that dural fistulae are formed after rupture of one or more sclerotic dural arteries that normally pass through the CS or rupture of CS veins receiving excess in-flow due to spontaneous thrombosis in collateral veins^(5)^. Fistulae may also result from a ruptured aneurism, and when high blood output is present, these lesions induce more severe signs and symptoms^(1,4)^. In the present case, however, initial symptoms were nonspecific, while more suggestive signs such as orbital murmur and fremitus were absent. Further, despite signs suggestive of orbital impairment, tomography did not reveal any abnormalities. Collectively, these initial findings may have contributed to delayed diagnosis. CCF was added to the differential diagnosis only after ocular changes worsened and neurological manifestations emerged. Rupture of the aneurysm may have led to intracranial hypertension, resulting in tonic-clonic seizures and worsening of the ocular signs and symptoms, including posterior venous stasis and increased IOP, which may have contributed to the observed exacerbation of corneal edema and hyphema^(6,7)^.

Thus, in cases of palpebral edema with signs of orbital involvement, especially among older adults, it is of fundamental importance to consider vascular abnormalities in the differential diagnosis and to perform appropriate tests. Auscultation of a murmur or fremitus’ palpation may, eventually, change the disease course. If CCF is suspected, it is mandatory to perform angiotomography or angioresonance imaging^(8)^. When doubts arise, it is imperative to perform cerebral angiography for diagnostic confirmation since the passage of an endovascular catheter permits the simultaneous identification, classification, and therapeutic management of the fistula through chemical or physical mechanisms (endoprostheses)^(2,9,10)^. The endovascular approach is the therapy of choice for high output fistulae. Depending on the type of fistula, other approaches may include clinical observation, carotid compression, surgery, or radiotherapy^(9,10)^. In most cases, the prognosis is favorable, with recovery during the first few days after flow interruption and a low rate of relapse and complications^(1,10)^.

We have found no previous reports on corneal edema, hematic impregnation, or hyphema secondary to CCF as observed in this case. It is possible that these findings may be associated with the severity of fistula (e.g., IOP)^(11,12)^. When present, these signs may help in the differential diagnosis between orbital cellulitis and CCF^(12)^.

This case illustrates the fundamental importance of considering more severe vision-threatening etiologies when conducting ophthalmological and follow-up examinations for patients presenting with severe progressive palpebral hyperemia and edema.
